# Multimodal lifestyle intervention using a web-based tool to improve cardiometabolic health in patients with serious mental illness: results of a cluster randomized controlled trial (LION)

**DOI:** 10.1186/s12888-019-2310-5

**Published:** 2019-11-05

**Authors:** Anne Looijmans, Frederike Jörg, Richard Bruggeman, Robert A. Schoevers, Eva Corpeleijn

**Affiliations:** 1 Department of Health Psychology, University Medical Center Groningen, Hanzeplein 1, PO Box 30.001, 9700 RB Groningen, The Netherlands; 2 Rob Giel Research Centre, University of Groningen, University Medical Center Groningen, Groningen, The Netherlands; 3 Research Department, Friesland Mental Health Services, Leeuwarden, The Netherlands; 4 Department of Psychiatry, University of Groningen, University Medical Center Groningen, Groningen, The Netherlands; 5 Department of Epidemiology, University of Groningen, University Medical Center Groningen, Groningen, The Netherlands

**Keywords:** Serious mental illness, Metabolic syndrome, Healthy lifestyle, Motivational interviewing, Health behaviour change, E-health, Physical activity

## Abstract

**Background:**

Unhealthy lifestyle behaviours contribute to alarming cardiometabolic risk in patients with serious mental illness (SMI). Evidence-based practical lifestyle tools supporting patients and staff in improving patient lifestyle are lacking.

**Methods:**

This multi-site randomized controlled pragmatic trial determined the effectiveness of a twelve-month multimodal lifestyle approach, including a web-based tool to improve patients’ cardiometabolic health, versus care-as-usual. Using the web tool, nurses (trained in motivational interviewing) assisted patients in assessing their lifestyle behaviours, creating a risk profile and constructing lifestyle goals, which were discussed during fortnightly regular care visits. Twenty-seven community-care and sheltered-living teams were randomized into intervention (*N* = 17) or control (*N* = 10) groups, including 244 patients (140 intervention/104 control, 49.2% male, 46.1 ± 10.8 years) with increased waist circumference (WC), BMI or fasting glucose. The primary outcomes concerned differences in WC after six and twelve months intervention, while BMI and metabolic syndrome Z-score were secondary outcome measures.

**Results:**

General multilevel linear mixed models adjusted for antipsychotic medication showed that differences in WC change between intervention and control were − 0.15 cm (95%CI: − 2.49; 2.19) after six and − 1.03 cm (95%CI: − 3.42; 1.35) after twelve months intervention; however, the differences were not statistically significant. No intervention effects were found for secondary outcome measures. The intervention increased patients’ readiness to change dietary behaviour.

**Conclusion:**

A multimodal web-based intervention facilitating nurses to address lifestyle changes in SMI patients did not improve patient cardiometabolic health. Web-tool use was lower than expected and nurses need more lifestyle coaching knowledge and skills. The type of intervention and delivery mode need optimization to realize effective lifestyle care for SMI patients.

**Trial registration:**

Dutch Trial Registry, www.trialregister.nl, NTR3765, 21 December 2012.

## Background

Among people with a serious mental illness (SMI), such as schizophrenia or other psychotic or bipolar disorders, the prevalence of obesity is 45–55%, while 10–15% have type 2 diabetes [1], which is almost four times higher than in the general population. Lifestyle interventions have proven to be effective in improving such cardiometabolic risk factors [2–4]. Most lifestyle interventions are relatively short and intense programmes that usually have at least three key components: exercise, diet and behavioural therapy [3, 4]. Although these interventions are effective in the short term, the benefits are seldom sustained in the long term. The literature shows that while interventions aimed at making small but sustainable changes in lifestyle behaviour – the ‘small steps approach’ – only lead to small weight changes in the short term, they prevent weight regain and result in more structural weight loss in the long term because such small changes can be sustained [5–7].

The small-steps approach is of interest to clinical care, insofar as it might be easier to implement in the daily care of patients than other interventions. It may also be suitable for any patient and not just those who are highly motivated and stable. For patients who are already motivated to change their behaviour, behavioural therapy strategies such as goal setting, making action plans and self-monitoring tend to work well [8]. It is known that unless motivational aspects are addressed explicitly, carrying out lifestyle interventions in a vulnerable and less motivated population will not lead to health improvements [9].

One effective approach to deal with unmotivated patients or those who are not ready to make changes is motivational interviewing (MI), developed by Miller and Rollnick [10], and including the stages-of-change construct from the Transtheoretical Model of Prochaska and DiClemente [11]. MI is a patient-centred counselling approach that targets behaviour change by strengthening a person’s own motivation and commitment using the ambivalence between their goals and behaviour. MI is effective in targeting lifestyle changes, such as improving weight status, Body Mass Index (BMI) and cholesterol levels of overweight and obese adults, and of clients in a broad range of other domains [12–14]. According to the stages-of-change model, a patient’s level of self-efficacy and readiness to change is reflected in one of five stages of change [15, 16]: precontemplation, contemplation, preparation, action and maintenance. These stages range from ‘no intention to change’ to ‘maintenance of behaviour change’. Treatment (or intervention) should adapt to a patient’s stage of change for maximum uptake of an intervention [15], for example creating awareness in the precontemplation stage and increasing intrinsic motivation in the preparation phase. MI is used to guide the patient’s transition from one stage to the other.

New technologies offer new opportunities for intervention. E-Health and m-Health technology has proven to be feasible and acceptable in the SMI patient population with regard to illness self-management and relapse prevention, adherence to medication, psycho-education and symptom monitoring [17]. To date, these technologies have rarely been used to address lifestyle behaviours in this patient population [17, 18], although results of pilot studies are emerging [19].

The current study presents a multimodal lifestyle tool called the Traffic Light Method, which is used to assist patients in improving their lifestyle. The Traffic Light Method is a multimodal tool, including a health assessment (‘visual risk profile’), tools for goal setting, (self-)monitoring of progress and informative text blocks based on lifestyle guidelines. Mental health (MH) nurses using the web tool were also trained in motivational interviewing and the stages-of-change model to be able to promote behaviour change in SMI patients [12, 20]. A three-month pilot study showed that the intervention was effective and feasible: patients receiving the multimodal lifestyle intervention (*N* = 20) on average lost three kilograms of body weight, performed more physical activity and rated their general well-being higher compared to usual-care controls (N = 20) [21].

The primary aim of the pragmatic Lifestyle Interventions for Outpatients with serious mental illness in the Netherlands (LION) trial was to study the effectiveness of a twelve-month multimodal lifestyle approach using the Traffic Light Method in terms of decreasing or stabilizing waist circumference (WC) after six and twelve months intervention, compared to care-as-usual. The secondary aim was to study the intervention effect on cardiometabolic risk. Cardiometabolic risk factors include Body Mass Index (BMI) and metabolic syndrome. The latter is a cluster of risk factors related to cardiovascular disease: diabetes and prediabetes, abdominal obesity, high blood pressure and elevated blood lipids (cholesterol and triglycerides). We hypothesized that the intervention would lead to stabilized or reduced WC, BMI and metabolic syndrome.

## Method

The LION study protocol has already been published [22]: it is a pragmatic single-blind multi-site cluster randomized controlled trial. The Medical Ethical Committee of the University Medical Center Groningen approved the study. Eligible patients received an information letter and signed informed consent before participating in the trial. The study was performed in accordance with the Declaration of Helsinki and registered in the Dutch Trial Registry (NTR3765, www.trialregister.nl, 21 December 2012). The trial adheres to the CONSORT guidelines [23] and the CONSORT-EHEALTH checklist (V.1.6.1) [24].

### Participants, recruitment and randomization

SMI patients treated by 21 Flexible Assertive Community Treatment (F-ACT) teams [25] and eight sheltered facility teams of five mental health organizations in the northern Netherlands (catchment area 3.6 million inhabitants) were invited to participate in the study within twelve months after the inclusion of the teams (January 2014 to October 2015). F-ACT teams offer outreach mental health care to community-dwelling patients in their own living environment, ranging from low intensive support to high intensive treatment [25]. When independent living is not possible, patients may reside in sheltered facilities in the community where they are supported in budgeting and other independent living skills. The F-ACT teams were clustered based on organization, caseload size, patient mean age, mean duration of patient admission, most prevalent diagnosis and location (urban or rural), and were randomized equally between intervention or control group. Randomization within eight blocks of two to three teams was performed using a random number generator by a member of the research team (FJ) not involved in the training of staff or the recruitment of patients. To avoid spill-over effects of the intervention, sheltered housing teams which relied on F-ACT teams for their patients’ mental health care, were assigned to the same condition as the F-ACT teams. In some teams, all of the nurses participated, while in others the team leader selected nurses.

MH nurses invited SMI patients to participate if their annual physical screening revealed at least one of the following metabolic risk factors: WC > 88/102 cm (females/males); fasting glucose > 5.6 mmol/L or HbA1c > 5.7% or > 39 mmol/mol; BMI > 25 kg/m^2^. Exclusion criteria were pregnancy, BMI < 19 kg/m^2^, or impairment in performing physical activity. Patients received an information letter and provided written informed consent. In total, with an alpha = 0.05 and power of 0.80, 275 patients were required to detect a clinically relevant reduction of 5.8 cm in the primary outcome for WC [26], taking into account 10% dropout.

### Intervention

The twelve-month multimodal, patient-centred lifestyle intervention was delivered by MH nurses, and included the use of the web tool ‘Traffic Light Method for Somatic Screening and Lifestyle’ (see below). MH nurses coached patients by using the web tool during regular care visits, ideally once every two weeks. Key features of the intervention were: [1] patient readiness for behaviour change was not a prerequisite for starting the intervention, [2] patients decided what lifestyle behaviour would be targeted and created their own lifestyle plan, [3] the intervention addressed diet and physical exercise and incorporated behavioural techniques, [4] active support from friends and family was incorporated into the lifestyle plan, and [5] nurses focused on coaching the patient and creating a healthier environment in the mental health care institution, if applicable. The patients’ level of readiness to change their diet and physical activity behaviours was assessed in the web tool, so MH nurses could better tailor the content of the intervention to the stages of change.

Nurses received one day of training on: (a) basic components of motivational interviewing [10] and the stage-of-change model [11], (b) side effects of psychotropic medication, (c) lifestyles of and risks for SMI patients, (d) working with the web tool Traffic Light Method, and (e) environmental factors that may influence effectively working with the Traffic Light Method (e.g. the availability of unhealthy products in the home environment). In addition, the study protocol was explained. After three months, nurses attended an evaluation session to discuss obstacles in the use of the tool, obstacles in motivating patients to participate and to recollect the study protocol. Due to the nature of the intervention, the trained LION nurses were not blind for study allocation.

Patients in the control condition received care-as-usual, which entails an annual Routine Outcome Monitoring (ROM) assessment, of which results are discussed with patients. They are referred to their GP when ROM results indicate this is necessary. Lifestyle guidance is more or less provided when patients express an interest.

### The web tool ‘Traffic Light Method for Somatic Screening and Lifestyle’

The Traffic Light Method was developed as a practical tool for use by nurses and patients in a Dutch mental health care organization and further advanced by a small spin-off company (Charly Green, Bilthoven, the Netherlands). The Traffic Light Method was constructed based on national and international guidelines for a healthy diet and physical activity and on the literature on somatic screening and lifestyle coaching, and was adapted after two rounds of Delphi panel expert discussion [21]. The tool provides knowledge and incorporates behavioural techniques to elicit behavioural change, such as creating awareness, goal setting, providing feedback and self-management. The tool addresses the lifestyle themes of diet, physical activity, medication use, personal hygiene, stressors, substance use and sexual behaviour.

The tool consists of a screening phase and a follow-up phase. In the screening phase, patients and nurses appraise the patient’s lifestyle behaviours. The Traffic Light Method generates a visual risk profile for each lifestyle behaviour based on the level of risk, represented by green (healthy), orange (medium-healthy) or red (unhealthy) traffic lights. Based on the lifestyle screening, patients construct a lifestyle plan with SMART (specific, measurable, attainable, realistic and timely [27]) behavioural goals. During the follow-up phase, nurses and patients evaluate the patient’s progress in achieving the lifestyle goals using follow-up reports during fortnightly regular care visits for approximately 15 min. After six months, patients and nurses screen lifestyle behaviours again, revisit and adjust or create a new lifestyle plan and evaluate this plan for the next six months until the intervention ends. In our study, it was estimated that patients would complete 23 follow-up reports over 12 months, i.e. 26 fortnightly visits minus the visits for the three lifestyle screening sessions. For a detailed description of the Traffic Light Method, see [21, 22].

### Measurements and outcomes

The primary outcome concerned waist circumference (WC; cm) after six and twelve months intervention. Secondary outcomes were measured by Body Mass Index (BMI; kg/m^2^) and metabolic syndrome Z-score (MS Z-score; SD), a standardized score for the cluster of five cardiometabolic risk factors. Information on age, sex, diagnosis and medication use was derived from patient record forms. As part of standard care, trained ROM nurses screen patients annually on physical and psychosocial outcomes according to protocol [28]. The data were used for baseline and twelve-month measurements. For the additional physical exam and lab test after six months of intervention (six-month measurement), participants received a nominal remuneration (EUR 5/USD 5.45). ROM nurses were blinded to treatment allocation.

WC, weight, height, systolic and diastolic blood pressure (BP) were measured according to protocol [22]. Fasting blood samples were collected in a hospital or other laboratory for levels of lipids (total cholesterol, LDL-cholesterol, HDL-cholesterol and triglycerides) and glucose metabolism (glucose, HbA1c). If not fasting, this was routinely indicated on the form.

Metabolic syndrome (MS) was defined as the presence of three or more of the following criteria [29]: WC ≥ 88/102 cm (female/male); systolic BP ≥ 130 and/or diastolic BP ≥ 85 mmHg or receiving antihypertensive medication; HDL-C < 1.03/1.3 mmol/L (female/male) or receiving lipid-lowering medication*;* fasting triglycerides ≥1.7 mmol/L or receiving lipid-lowering medication; and fasting glucose ≥6.1 mmol/L [30] or receiving antihyperglycemic medication. When fasting glucose levels were not available, patients were considered to fulfil the glucose risk criterion if they reported to have diabetes or if HbA1c ≥ 42.0 mmol/mol [31]. Since the dichotomization of the MS components reduces sensitivity to changes over time, the individual components were standardized into Z-scores (with HDL-cholesterol Z-score multiplied by − 1) [32, 33] and the sum, divided by five, was used as a continuous variable for the degree of metabolic syndrome (MS Z-score). BP was standardized using mean arterial pressure (MAP).

Antipsychotic medication (AP) was categorized into three groups according to the strength of its side effect on cardiometabolic health (none, mild or strong) based on the literature [34, 35] (see e Additional file 1 Table S1).

Patient readiness to change physical activity or dietary behaviour was assessed by a question representing the five phases of the stage-of-change model [11]. Answers ranged from ‘not willing to change within six months’ (precontemplation), ‘willing to change within six months’ (contemplation), ‘willing to change within one month’ (preparation), ‘consider myself acting healthily for less than six months’ (action) to ‘consider myself acting healthily for more than six months’ (maintenance phase).

The number of follow-up reports in the Traffic Light Method web tool represents the level of adherence to the intervention.

### Analyses

Results were presented as means (95% confidence interval) or medians (25-75th percentile). Data were analysed using SPSS version 22 [36], with a *p*-value of 0.05 considered statistically significant. The intervention effect was tested using an intention-to-treat approach with a multi-level, subject-specific linear mixed model that had an unstructured variance structure, controlling for teams to adjust for clustering of patients within teams and adjusting for type of AP medication. Intervention effects were tested in stratified analyses of pre-specified subgroups based on sex, age and type of housing. In explorative per-protocol analyses, adhering participants (high users) were compared to the control group using the same linear mixed models as described above. Participants who completed at least one lifestyle behaviour screening, constructed lifestyle goals and completed ten or more follow-up reports were considered high users. We also compared the percentage of participants who improved or deteriorated by ≥5% in WC or BMI after six and twelve months between intervention and control groups using Chi-square tests.

We also used a Chi-square test to determine the intervention effects on patient readiness to change dietary or physical activity behaviour by comparing the percentage of intervention participants who had shifted towards more readiness to change between baseline, six and twelve months to the percentage of participants in the control group who had done so.

## Results

**Fig. 1:**
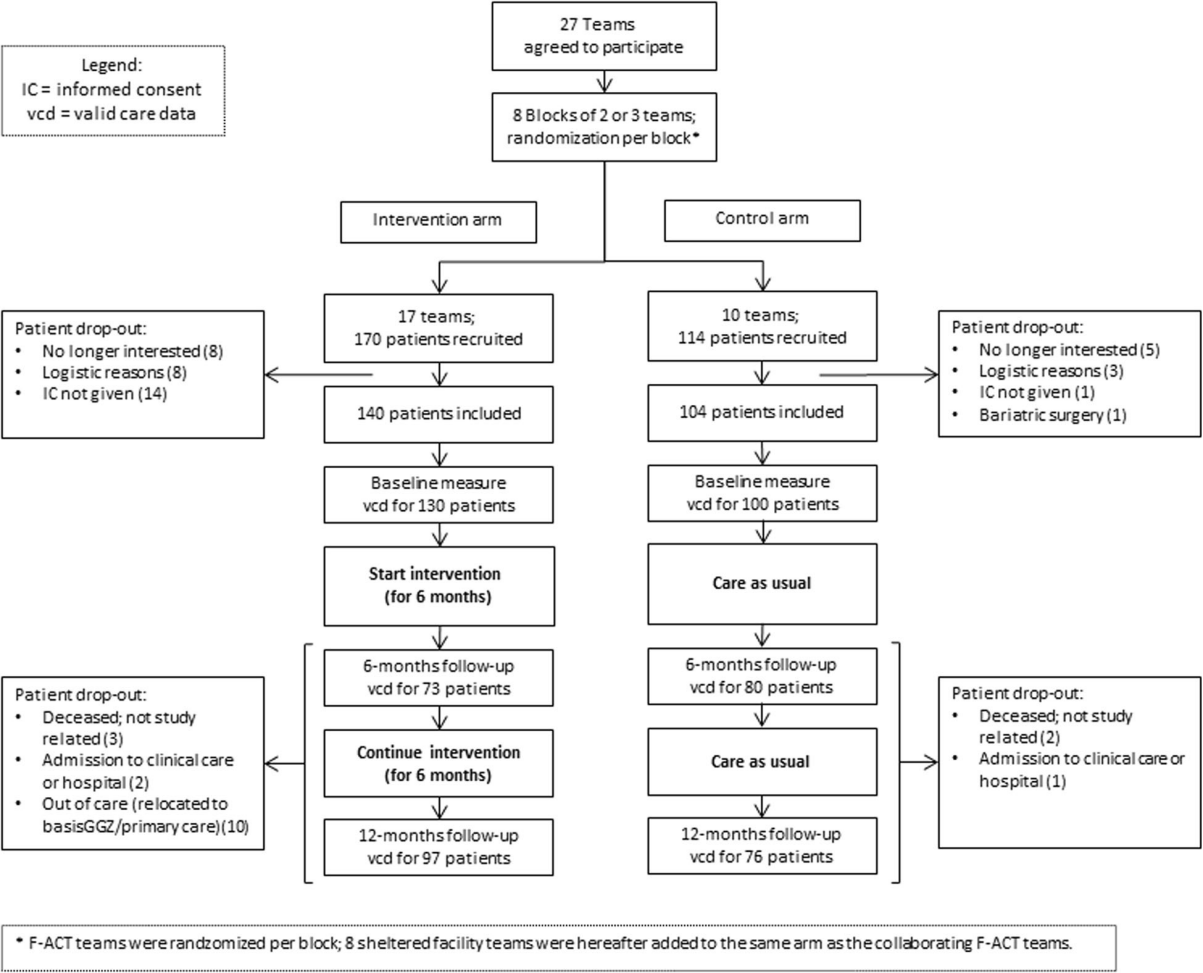
Flow chart of patients in the LION trial

In total, 244 patients (140 intervention; 104 control) were included in the trial, of whom 49.2% were male and the mean age was 46.1 ± 10.8 years (Table 1). Patients in the intervention group were on average 4.3 years younger (*p* = .002) and had a higher BMI (*p* = .045) than patients in the control group. More teams ended up in the intervention (*N* = 17) than in the control group (*N* = 10) due to large reorganizations that took place in mental health care during the first phase of the trial. This led to teams being combined, split or disbanded after the randomization procedure had been completed (Fig. 1) [22].

**Table 1:** Baseline characteristics of LION study participants

	N	Total	Intervention group	Control group	*p*-values
General information					
Teams, N		27	17	10	
Nurses, N		138	82	56	
Patient characteristics					
Patients, N		244	140	104	
Age, mean ± SD, years	240	46.1 ± 10.8	44.3 ± 10.9	48.6 ± 10.2	**.002**
Male sex, N (%)		120 (49.2)	66 (47.1)	54 (51.9)	.46
Housing, N	240				.38
F-ACT teams (patients)		19 (193)	12 (108)	7 (85)	
Sheltered living teams (patients)		8 (51)	5 (32)	3 (19)	
Years since first contact MH organisation, mean ± SD, years	220	17.0 ± 11.0	15.6 ± 11.3	19.0 ± 10.3	**.021**
Adiposity					
Waist circumference, mean ± SD, cm					
- male	114	111.3 ± 12.7	112.3 ± 14.2	110.0 ± 10.7	.32
- female	116	110.2 ± 16.3	111.9 ± 17.0	107.8 ± 15.0	.18
Body Mass Index (BMI), mean ± SD, kg/m^2^	233	32.0 ± 6.4	32.7 ± 7.2	31.1 ± 5.1	**.045**
BMI categories, N (%):	233				.36
Normal (BMI < 25)		21 (9.0)	11 (8.3)	10 (10.0)	
Overweight (25 ≤ BMI < 30)		81 (34.8)	44 (33.1)	37 (37.0)	
Obese I (30 ≤ BMI < 35)		70 (30.0)	40 (30.1)	30 (30.0)	
Obese II (35 ≤ BMI < 40)		36 (15.5)	19 (14.3)	17 (17.0)	
Obese III (BMI ≥40)		25 (10.7)	19 (14.3)	6 (6.0)	
Blood pressure (BP), mean ± SD, mmHG					
Systolic BP	230	133.1 ± 17.0	132.9 ± 17.3	133.4 ± 16.7	.82
Diastolic BP	227	84.1 ± 10.5	85.0 ± 10.5	82.9 ± 10.5	.15
Use of BP lowering medication, No. (%)	171	45 (26.3)	21 (22.1)	24 (31.6)	.16
Lipids					
Total cholesterol, mean ± SD, mmol/L	199	5.08 ± 1.11	5.17 ± 1.05	4.96 ± 1.18	.20
HDL-cholesterol, mean ± SD, mmol/L					
- male	107	1.03 ± 0.23	1.01 ± 0.23	1.05 ± 0.22	.38
- female	103	1.36 ± 0.47	1.35 ± 0.53	1.36 ± 0.37	.95
LDL-cholesterol, mean ± SD, mmol/L	196	3.07 ± 0.94	3.09 ± 0.88	3.05 ± 1.02	.75
Triglycerides, median [25-75th %], mmol/L	94	1.73 [1.08; 2.41]	1.68 [1.03; 2.53]	1.76 [1.22; 2.15]	.90
Use of lipid lowering medication, No. (%)	171	45 (26.3)	22 (22.7)	23 (31.1)	.22
Glucose metabolism					
Fasting glucose, median [25-75th %], mmol/L	93	6.0 [5.4; 7.0]	5.7 [5.3; 7.0]	6.2 [5.7; 7.0]	.09
HbA1c, median [25-75th %], (%)	190	36.0 [33.3; 41.0]	36.0 [33.0; 39.0]	38.0 [34.0; 44.0]	**.009**
Diagnosis of diabetes^b^	235	73 (31.1)	36 (27.1)	37 (36.3)	.13
Use of glucose lowering medication, N (%)	162	37 (22.8)	17 (18.5)	20 (28.6)	.13
Metabolic syndrome, N (%)	84	56 (66.7)	25 (56.8)	31 (77.5)	.37
Metabolic syndrome Z-score^a^, mean ± SD, SD	84	0.65 ± 0.92	0.61 ± 0.96	0.69 ± 0.88	.68
Psychiatric characteristics					
Psychiatric diagnosis, N (%)	243				
Psychotic disorder		140 (57.6)	86 (61.4)	54 (52.5)	.16
Mood disorder		68 (28.0)	36 (25.7)	32 (31.1)	.36
Personality disorder		64 (26.3)	34 (24.3)	30 (29.1)	.40
Anxiety disorder		33 (13.6)	18 (12.9)	15 (14.6)	.70
Psychiatric comorbidity^c^, N (%)	243	75 (30.9)	40 (28.6)	35 (34.0)	.37
Use of antipsychotics, N (%)	217	187 (86.2)	108 (87.8)	79 (84.0)	.43
Antipsychotic medication based on metabolic side effect^d^, N (%)	224				.74
No effect		71 (31.7)	42 (33.1)	29 (29.9)	
Medium effect		76 (33.9)	44 (34.6)	32 (33.0)	
High effect		77 (31.7)	41 (32.3)	36 (37.1)	
Smoking, yes, N (%)	198	110 (55.6)	60 (55.6)	50 (55.6)	.99
Stage of change^e^, N (%)					
Dietary behaviour	209				.13
Pre-contemplation phase		11 (5.3)	5 (4.3)	6 (6.5)	
Contemplation phase		51 (24.4)	29 (24.8)	22 (23.9)	
Preparation phase		56 (26.8)	39 (33.3)	17 (18.5)	
Action phase		21 (10.0)	11 (9.4)	10 (10.9)	
Maintenance phase		70 (33.5)	33 (28.2)	37 (40.2)	
Physical activity behaviour	201				**.00**
Pre-contemplation phase		29 (14.4)	12 (11.0)	17 (18.5)	
Contemplation phase		49 (24.4)	36 (33.0)	13 (14.1)	
Preparation phase		43 (21.4)	29 (26.6)	14 (15.2)	
Action phase		18 (9.0)	10 (9.2)	8 (8.7)	
Maintenance phase		62 (30.8)	22 (20.2)	40 (43.5)	

*Note*: SI conversion factors: to convert total cholesterol, HDL-cholesterol and LDL-cholesterol to mg/dL, divide values by 0.0259; to convert triglycerides to mg/dL, divide values by 0.0113; to convert fasting glucose to mg/dL, divide values by 0.0555. Baseline differences were tested with Student’s T, Mann Whitney U or Chi square tests. Bold *p*-values denote statistical significance at the *p* < 0.05 level

a The means and standard deviations (SD) of the patients ranging within healthy reference values were used to standardize HDL-C (1.1–2.0 mmol/L in female and 0.9–1.7 mmol/L in male patients), triglycerides (≤ 2.2 mmol/L) and fasting glucose (≤ 7.1 mmol/L) or HbA1c (< 8.0%)

b Diabetes was defined based on reported diagnosis of diabetes, use of antihyperglycemic medication, fasting glucose ≥7.1 mmol/L or HbA1c ≥ 48 mmol/mol

c Two or more of the defined diagnoses

d If no antipsychotic medication was used, this was categorized as the no effect group

e Pre-contemplation phase ‘I am eating (a little bit) unhealthily and do not intend to eat healthily in six months’; Contemplation phase ‘I am eating (a little bit) unhealthily and intend to eat healthily in six months’; Preparation phase ‘I am eating (a little bit) unhealthily and intend to eat healthily in one month’; Action phase ‘I have eaten healthily for less than six months’; Maintenance phase ‘I have eaten healthily for more than six months’. For stage of change for physical activity behaviour, eating was replaced by activity (e.g. ‘My activity is (a little bit) unhealthy and I do not intend to act healthily in six months’, etc.)

**Fig. 2:**
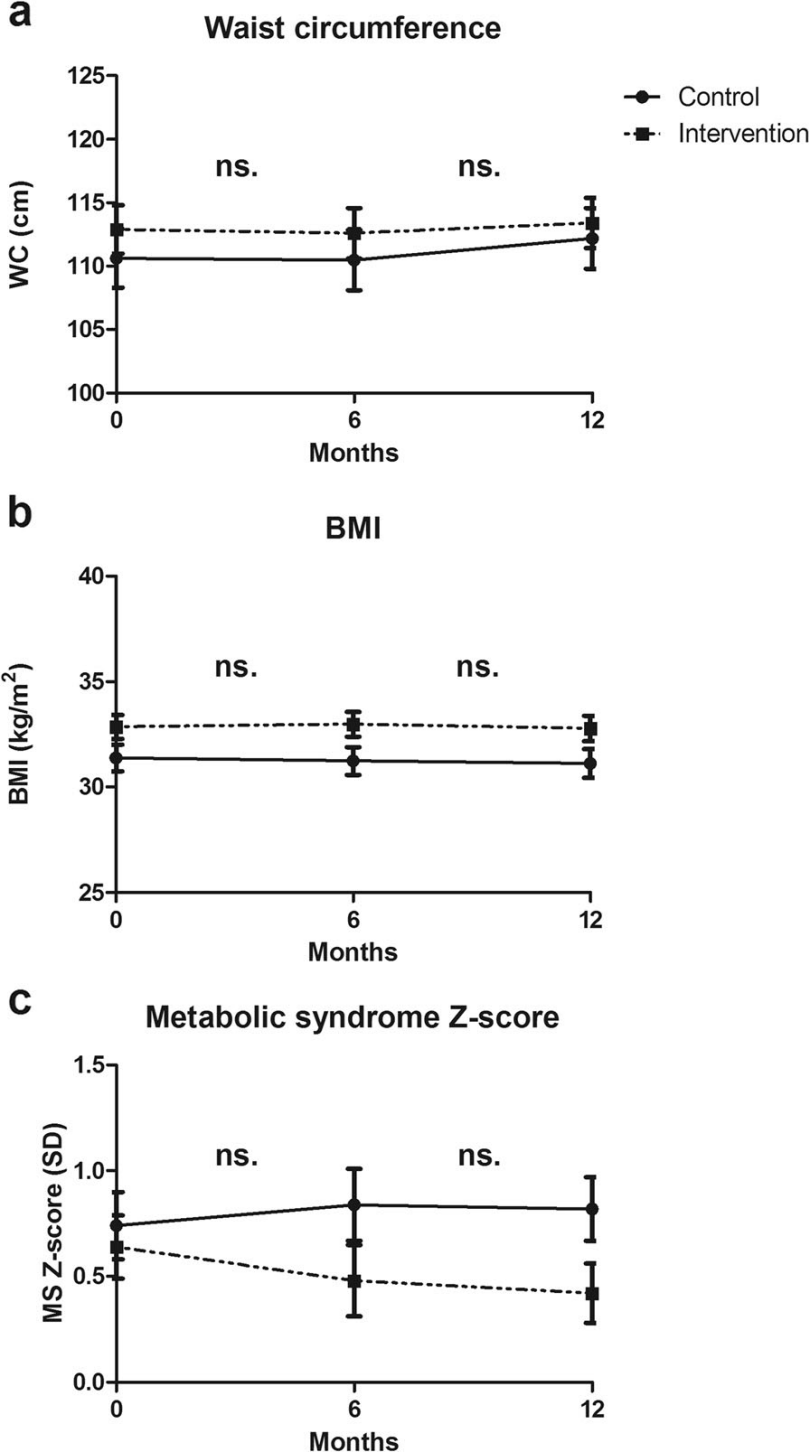
Somatic outcomes at baseline, six and twelve months per condition. Legend: Estimated marginal means and standard errors for: **a**) waist circumference, **b**) BMI and **c**) metabolic syndrome Z-score for intervention and control groups at baseline, six and twelve months.

Results for the intervention and control groups over time are presented in Fig. 2 and e Additional file 2 Table S2. In intention-to-treat analyses, WC did not significantly differ from the control group after six (-0.15 cm (-2.49; 2.19)) and after twelve months (-1.03 cm (-3.42; 1.35)) (Table 2). ForBMI and MS Z-score, no significant differences were found either. Compared to the control group, no significant intervention effects on WC, BMI or MS Z-score were found for males vs females, for younger (≤ 46.0 years) vs older (> 46.0 years) participants or for F-ACT vs sheltered housing participants (e Additional file 3 Table S3). No differences between the intervention and control groups were found regarding the percentage of participants who improved or deteriorated by ≥5% in WC or BMI after six or twelve months.

**Table 2:** Somatic outcomes after six and twelve months of lifestyle intervention in SMI patients. Results of general linear mixed models analyses with adjustment for AP medication side effect

	Waist circumference (*N* = 238)			BMI (*N* = 240)			Metabolic syndrome Z-score (*N* = 115)		
	β	95% CI	*p*-value	β	95% CI	*p*-value	β	95% CI	*p*-value
Intervention effect^a^									
at 6 months^b^	−0.15	[− 2.49; 2.19]	.90	0.27	[−0.32; 0.85]	.38	−0.25	[− 0.69;0.18]	.24
at 12 months^b^	−1.03	[−3.42; 1.35]	.39	0.18	[−0.49; 0.86]	.60	−0.30	[−0.66; 0.05]	.09
Group difference (intervention vs control)	2.26	[−3.91; 8.44]	.45	1.47	[−0.17; 3.11]	.08	−0.10	[−0.54; 0.34]	.63
Time effect only									
6 months	−0.15	[−1.84; 1.54]	.86	−0.15	[−0.57; 0.27]	.49	0.10	[−0.19; 0.40]	.48
12 months	1.56	[−0.23; 3.34]	.08	−0.26	[−0.77; 0.24]	.31	0.08	[−0.17; 0.33]	.51
AP med. Side effect^c^									
Medium	1.99	[−0.91; 4.90]	.18	0.90	[−0.01; 1.81]	.05	−0.07	[−0.51; 0.36]	.74
High	0.17	[−3.04; 3.38]	.92	0.08	[−1.09; 1.25]	.90	0.40	[0.00; 0.80]	**.049**

Abbreviations: AP medication side effect: antipsychotic medication side effect on metabolism; CI: confidence interval.

^a^ control group is reference

^b^ group x time

^c^ no AP medication side effect is reference

Bold *p*-values denote statistical significance at the *p* < 0.05 level

**Fig. 3:**
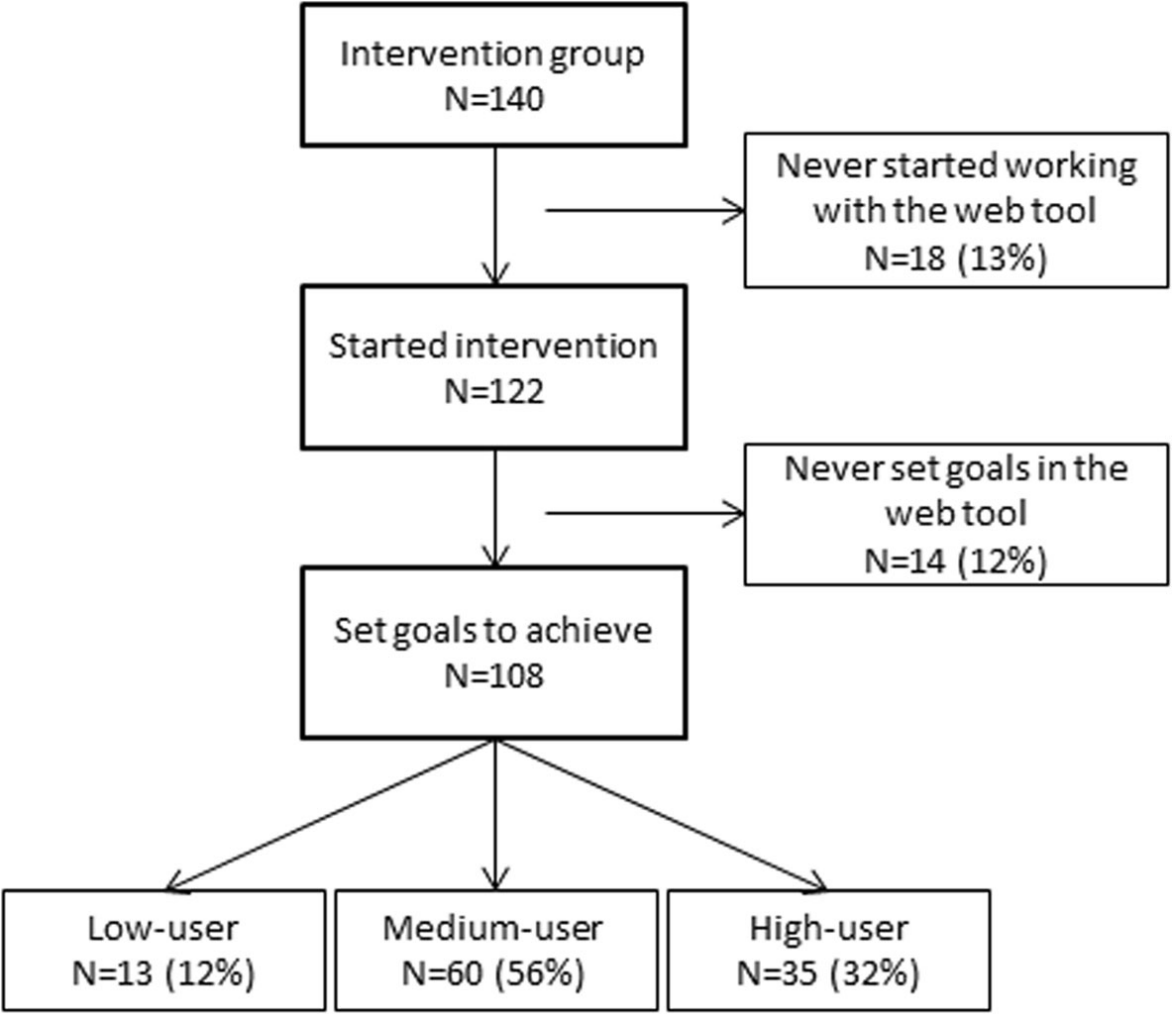
Intervention adherence of patients in the LION trial. Legend: If participants completed at least one lifestyle behaviour screening and constructed a lifestyle plan with lifestyle goals, they were considered a low user when no follow-up reports were completed; a medium user when between one and nine follow-up reports were completed; and a high user when ten or more follow-up reports were completed.

The use of the web tool during regular care visits was much lower than the estimated 23 follow-up reports. In the intervention group, 108 of all 140 (77%) patients completed at least one lifestyle behaviour screening and constructed subsequent lifestyle plans with lifestyle goals (Fig. 3). Of those, low users (*N* = 13; 12%) had no follow-up reports, while medium users (*N* = 60; 56%) and high users (*N* = 35; 32%) had medians of 4.0 [2.3; 7.0] and 14.0 [11.0; 18.0] follow-up reports, respectively. Patients primarily constructed lifestyle goals related to diet (*N* = 141; 41.7%), physical activity (*N* = 83; 24.6%) or a combination of both (*N* = 37; 10.9%), but also related to smoking (*N* = 17; 5.0%) and sleeping behaviours (*N* = 15; 4.4%). At baseline or second measurement, almost all intervention patients (*N* = 99; 92%) set at least one goal related to energy intake or expenditure. Explorative analyses were conducted in the high-user group: WC change was − 1.87 cm (− 7.31; 1.56) after six and − 1.69 cm (− 4.96; 1.58) after twelve months of intervention compared to controls, although this was not statistically significant. BMI and MS Z-score did not differ over time in high users compared to controls.

At baseline, the readiness to change physical activity behaviour differed significantly between the intervention group and control group: 48 (52.2%) control patients considered themselves healthy for less or more than six months with regard to physical activity compared to 32 (29.4%) patients in the intervention group (*p* < .001) (Table 1). Although the intervention group thus had more opportunity to increase readiness to change, over time, no significantly different changes in stage of change were found between the groups. With regard to dietary stage of change at baseline, no differences were found between the intervention and control groups. After six months of intervention, more patients in the intervention group increased in their readiness to change their dietary behaviours (40% vs 23%) and fewer decreased in readiness to change (19% vs 39%), when compared to the control group (*p* = .049). After twelve months, 40% of the patients in the intervention group increased and 26% decreased in their readiness to change their dietary behaviours, compared to a 20% increase and 29% decrease in the control group (*p* = .023).

## Discussion

Considering the alarming cardiometabolic risks for SMI patients, staff involved in regular mental health care practice urgently need tools and training to improve patient lifestyle. The twelve-month multimodal web-based intervention facilitating nurses in addressing SMI patient lifestyle change did not improve the patients’ cardiometabolic health but did improve the patients’ readiness to change their dietary behaviour. The study shows that the use of the web tool was lower than expected. The type of intervention and delivery mode will need optimization to realize effective lifestyle care for SMI patients.

The results of this trial can be compared to several recently published pragmatic trials in this field that have comparable sample sizes and follow-up duration [37–42]. The results of the trials are inconsistent; some report significant weight loss in the intervention group [37–39], while others yield findings comparable to ours [40–42]. This inconsistency may be due to a number of reasons, which are discussed below. In our view, a balance needs to be found, firstly in relation to the type of intervention offered: should a long-term patient-centred programme focused on counselling about lifestyle behaviour be employed, or a high-intensity short-term programme focused on supervised exercise and intensive diet? Secondly, it is important to determine who can participate in the lifestyle programme: should all patients be targeted or only those who are highly motivated and ready, or only those with high risk? Finally, there must be a balance in the delivery of the intervention: should it occur as part of care involving caregivers with whom the patient is already familiar, or does it require health professionals specifically trained to provide lifestyle care?

The outcomes of our trial may have been related to the type of intervention. In the literature, the more effective interventions are intensive and include guided exercise sessions [37–39], whereas those with null findings [40–42] are mainly oriented towards promoting behavioural change using counselling, including motivational techniques. This could imply that interventions based on counselling and education may not be sufficiently effective, and that guided exercise sessions, in addition to motivational interviewing techniques, need to be included to achieve health gains. However, we might also conclude that interventions that mainly focus on increasing motivation to change unhealthy behaviours need a longer follow-up period when studying health outcomes, since health gains should probably be expected much later when the nurses’ aim is to provide patients with support to reach personal goals, rather than organizing this for them. This is illustrated in our study by the fact that in the intervention group, readiness to change in diet significantly increased after twelve months. In addition, those who completed more sessions over a longer period of time lost more weight in the end than the control group, albeit not significantly.

The difference in effects that interventions have on body weight may partly be related to the type of patients enrolled in the studies. In the studies in which weight loss was achieved [37–39], patients knew beforehand that structured and guided exercise sessions were part of the intervention and, thus, it is most likely that they were highly motivated and willing to take part in such an intervention. One drawback of this strategy is that it may compromise external validity [43], as some studies have indeed reported [38, 39]. In contrast, while the STEPWISE trial reported having no trouble recruiting and retaining patients in the trial, intervention uptake was a challenge [41].

The findings may also be inconsistent because study participants may have differed in their baseline risk. The more effective trials included participants with a BMI ≥25 kg/m^2^, whereas the trials with null findings included patients with an interest in lifestyle (change) [40–42] or, in our case, meeting one of three inclusion criteria regarding metabolic risk. As a result, apart from the STEPWISE trial, mean baseline BMI levels were lower in the studies without effect. Naslund (2016) showed that a higher BMI at baseline is associated with more weight change upon intervention [44], supporting our observation.

With regard to the delivery of the intervention, we chose MH nurses, who included the intervention in their regular care contact sessions. The number of completed screenings and follow-up reports indicates that treatment adherence was lower than expected, suggesting that it may have been difficult for the nurses to carry out the intervention as part of their regular work. In the more effective trials [37–39], specially appointed professionals were allocated dedicated hours for lifestyle coaching and exercise guidance with the exclusive priority to improve patient lifestyle. In other words, lifestyle coaching requires specific knowledge, skills and dedicated time, and should probably be the responsibility of professional lifestyle coaches rather than an additional task of nurses.

In summary, regarding aspects of the type of intervention, patient inclusion and delivery of the lifestyle care, all approaches may differ with regard to the effectiveness in the short term, and in their respective advantages and disadvantages. More insight is needed into the long-term effects of these trials to warrant fair comparisons in terms of effectiveness, and to decide what strategies, or combinations of strategies, are best to provide effective lifestyle care for patients with a serious mental illness.

### Limitations

Several factors may have influenced the implementation and impact of the intervention. Large budget cutbacks in mental health organizations at the start of this trial resulted in an unexpected increased workload for MH professionals and the transfer of SMI patients to a more limited form of general mental health care. This may have negatively affected the MH nurses’ opportunity and motivation to implement the intervention [45] on the one hand, and may have caused loss to follow-up of probably the most stable patients, on the other hand. Furthermore, although most nurses had received MI training before, the MI skills of some nurses might have been insufficient to increase patients’ intrinsic motivation. Regretfully, we have no audio-recordings of the lifestyle sessions that would enable us to measure the level of fidelity in motivational interviewing techniques. However, of note in this respect is that the STEPWISE trial did not yield significant results, despite the reported high fidelity in motivational interviewing techniques [41]. Furthermore, filling in the follow-up reports was reported to take much longer than the expected fifteen minutes per regular care visit. Some nurses also experienced practical problems, such as no computer/laptop available or no access to the internet in rural areas.

## Conclusion

The Traffic Light Method is a multimodal tool, which includes a health assessment (‘visual risk profile’), tools for goal setting, (self-)monitoring of progress and informative text blocks based on lifestyle guidelines. Nurses using the web tool were trained in motivational interviewing and the stages-of-change model to be able to promote behaviour change in SMI patients. Using this tool did not improve cardiometabolic health. It did, however, improve the readiness to change dietary behaviour. Interventions mainly focusing on increasing motivation and readiness to change might need a much longer follow-up, as it takes time to translate intentions into actual behaviour change, especially in this vulnerable population. A comparison of our results with those in the existing literature suggests that both the type of intervention and the delivery mode need optimization to realize effective lifestyle care for SMI patients. In addition, we propose that lifestyle coaching for SMI patients be considered a complex specialization demanding specific knowledge and skills, and that it should thus not be one of the many tasks assigned to MH nurses, but rather the responsibility of appointed lifestyle professionals.

## Supplementary information

**Additional file 1: Table S1.** Categorization of antipsychotic medication according to the strength of the side effect (none, mild or strong) on cardiometabolic health.

**Additional file 2: Table S2.** Estimated marginal means and standard errors for waist circumference, BMI and metabolic syndrome (MS) Z-score for intervention and control groups at baseline, six and twelve months.

**Additional file 3: Table S3.** Waist circumference, BMI and metabolic syndrome Z-score after six and twelve months of lifestyle intervention in SMI patients stratified for gender, age group and type of facility. Results of linear mixed models analyses with adjustment for AP medication side effect.

## Data Availability

The datasets generated and/or analysed during the current study are not publicly available. However, as patients gave written consent to the sharing of their data for this study and could optionally give consent to the sharing of their data for future studies on mental health, they are available from the corresponding author on reasonable request.

## Abbreviations

AP: Antipsychotic medication
BMI: Body Mass Index
F-ACT: Flexible Assertive Community Treatment
MAP: Mean arterial pressure
MH: Mental health
MS Z-score: Metabolic syndrome Z-score
SMI: Serious mental illness
WC: Waist circumference
