# Supplementary material for: Multimodal lifestyle intervention using a web-based tool to improve cardiometabolic health in patients with serious mental illness: results of a cluster randomized controlled trial (LION)
Source: BMC Psychiatry. 2019 Nov 5;19:339. doi: 10.1186/s12888-019-2310-5 (PMC6833253; doi:10.1186/s12888-019-2310-5)
Supplement: Supplementary file 1 — Additional file 1: Table S1. Categorization of antipsychotic medication according to the strength of the side effect (none, mild or strong) on cardiometabolic health. [file 12888_2019_2310_MOESM1_ESM.doc]

**eTable 1. Categorization of antipsychotic medication according to the strength of the side effect (none, mild or strong) on cardiometabolic health**

**Categorizing is based on the Dutch Farmacotherapeutical Compass (FC), website UptoDate (UtD) and the expert opinion (EP) of three psychiatrists.**

| **Antipsychotic medication** | **Source** |
| --- | --- |
| **No cardiometabolic influence** | |
| Aripiprazole | FC |
| Haloperidol | FC |
| Bromperidol | EP |
| Flupenthixol | FC |
| Pimozide | FC |
| Sulpiride | FC |
| Tiapride | EP |
| Penfluridol | EP |
| Fluphenazine | UtD |
| **Mild cardiometabolic influence** | |
| Risperidone | FC |
| Quetiapine | FC |
| Chlorprothixene | EP |
| Levomepromazine | EP |
| Paliperidone | UtD |
| Periciazine | EP |
| Pipamperon | EP |
| Zuclopenthixol | EP |
| Fluspirilene | EP |
| **Strong cardiometabolic influence** | |
| Clozapine | FC |
| Olanzapine | FC |

Note: FC = (Dutch) National Health Care Institute (Zorginstituut Nederland). Farmacotherapeutisch kompas. http://www.farmacotherapeutischkompas.nl/inleidendeteksten/i/inl%20antipsychotica.asp. Retrieved 23 June 2015; UtD = Selected adverse effects of antipsychotic medications for schizophrenia (www.uptodate.com). Retrieved 3 Augustus 2015; EP = expert opinion.
