# Supplementary material for: Multimodal lifestyle intervention using a web-based tool to improve cardiometabolic health in patients with serious mental illness: results of a cluster randomized controlled trial (LION)
Source: BMC Psychiatry. 2019 Nov 5;19:339. doi: 10.1186/s12888-019-2310-5 (PMC6833253; doi:10.1186/s12888-019-2310-5)
Supplement: Supplementary file 2 — Additional file 2: Table S2. Estimated marginal means and standard errors for waist circumference, BMI and metabolic syndrome (MS) Z-score for intervention and control groups at baseline, six and twelve months. [file 12888_2019_2310_MOESM2_ESM.doc]

**eTable 2. Estimated marginal means and standard errors for waist circumference, BMI and metabolic syndrome (MS) Z-score for intervention and control group at baseline, six and twelve months**

|  | **N** | **Baseline** | **6 months** | **12 months** |
| --- | --- | --- | --- | --- |
| **Waist circumference (cm)** |  |  |  |  |
| Intervention | 135 | 112.9 ± 1.9 | 112.6 ± 2.0 | 113.4 ± 2.0 |
| Control | 103 | 110.6 ± 2.3 | 110.5 ± 2.4 | 112.2 ± 2.4 |
| **BMI (kg/m^2^)** |  |  |  |  |
| Intervention | 137 | 32.86 ± 0.56 | 32.98 ± 0.59 | 32.78 ± 0.60 |
| Control | 103 | 31.38 ± 0.64 | 31.24 ± 0.66 | 31.12 ± 0.68 |
| **MS Z-score (SD)** |  |  |  |  |
| Intervention | 58 | 0.64 ± 0.15 | 0.48 ± 0.17 | 0.42 ± 0.14 |
| Control | 57 | 0.74 ± 0.16 | 0.84 ± 0.17 | 0.82 ± 0.15 |
