# Supplementary material for: Multimodal lifestyle intervention using a web-based tool to improve cardiometabolic health in patients with serious mental illness: results of a cluster randomized controlled trial (LION)
Source: BMC Psychiatry. 2019 Nov 5;19:339. doi: 10.1186/s12888-019-2310-5 (PMC6833253; doi:10.1186/s12888-019-2310-5)
Supplement: Supplementary file 3 — Additional file 3: Table S3. Waist circumference, BMI and metabolic syndrome Z-score after six and twelve months of lifestyle intervention in SMI patients stratified for gender, age group and type of facility. Results of linear mixed models analyses with adjustment for AP medication side effect. [file 12888_2019_2310_MOESM3_ESM.doc]

**eTable 3. Waist circumference, BMI and metabolic syndrome Z-score after six and twelve months of lifestyle intervention in SMI patients stratified for gender, age group and type of facility. Results of linear mixed models analyses with adjustment for AP medication side effect**

|  | **WAIST CIRCUMFERENCE** | | | | | | | | | | | | | | | | | | |
| --- | --- | --- | --- | --- | --- | --- | --- | --- | --- | --- | --- | --- | --- | --- | --- | --- | --- | --- | --- |
|  | **Gender** | | | | | | **Age** | | | | | | | **Facility** | | | | | |
|  | Males  (n=119) | | | Females  (n=119) | | | ≤46 yrs  (n=120) | | | | >46 yrs  (n=118) | | | F-ACT  (n=189) | | | Sheltered  (n=49) | | |
|  | **β** | **95% CI** | ***p*** | **β** | **95% CI** | ***p*** | **β** | | **95% CI** | ***p*** | **β** | **95% CI** | ***p*** | **β** | **95% CI** | ***p*** | **β** | **95% CI** | ***p*** |
| Intervention effect^a^ |  |  |  |  |  |  |  | |  |  |  |  |  |  |  |  |  |  |  |
| at 3 months^b^ | 0.17 | -3.03; 3.38 | .91 | -0.63 | -4.10; 2.83 | .72 | -1.63 | | -5.17; 1.91 | .36 | 0.57 | -2.75; 3.88 | .73 | -0.20 | -2.91; 2.52 | .89 | -0.77 | -4.66; 3.12 | .69 |
| at 12 months^b^ | -1.04 | -3.86; 1.78 | .47 | -0.74 | -4.70; 3.22 | .71 | -1.13 | | -5.07; 2.80 | .57 | -2.09 | -5.06; 0.89 | .17 | -0.85 | -3.62; 1.91 | .54 | -2.27 | -6.53; 1.99 | .28 |
| Group difference (intervention vs control) | 2.75 | -4.91; 10.41 | .46 | 2.24 | -5.16; 9.64 | .53 | 4.86 | | -3.06; 12.79 | .21 | 1.53 | -3.65; 6.70 | .56 | 5.76 | -1.31; 12.83 | .10 | -7.39 | -20.94; 6.16 | .23 |
| Time effect only |  |  |  |  |  |  |  | |  |  |  |  |  |  |  |  |  |  |  |
| 3 months | -0.85 | -3.12; 1.43 | .46 | 0.75 | -1.82; 3.32 | .56 | 2.04 | | -0.79; 4.88 | .16 | -1.50 | -3.63; 0.62 | .16 | -0.60 | -2.52; 1.32 | .54 | 2.50 | -0.55; 5.54 | .10 |
| 12 months | 1.48 | -0.57; 3.52 | .15 | 1.66 | -1.38; 4.71 | .28 | 3.18 | | -0.03; 6.38 | .052 | 0.59 | -1.44; 2.61 | .57 | 1.24 | -0.81; 3.29 | .23 | 3.62 | 0.28; 6.96 | **.04** |
|  | **BMI** | | | | | | | | | | | | | | | | | | |
|  | **Gender** | | | | | | **Age** | | | | | | | **Facility** | | | | | |
|  | Males  (n=120) | | | Females  (n=120) | | | ≤46 yrs  (n=120) | | | | >46 yrs  (n=120) | | | F-ACT  (n=190) | | | Sheltered  (n=50) | | |
|  | **β** | **95% CI** | ***p*** | **β** | **95% CI** | ***p*** | **β** | | **95% CI** | ***p*** | **β** | **95% CI** | ***p*** | **β** | **95% CI** | ***p*** | **β** | **95% CI** | ***p*** |
| Intervention effect^a^ |  |  |  |  |  |  |  | |  |  |  |  |  |  |  |  |  |  |  |
| at 3 months^b^ | 0.27 | -0.53; 1.06 | .51 | 0.27 | -0.63; 1.17 | .56 | 0.03 | | -0.81; 0.87 | .94 | 0.27 | -0.63; 1.17 | .55 | 0.24 | -0.45; 0.94 | .49 | 0.31 | -0.65; 1.27 | .51 |
| at 12 months^b^ | 0.59 | -0.29; 1.48 | .19 | -0.11 | -1.15; 0.92 | .83 | 0.00 | | -1.09; 1.10 | .99 | 0.06 | -0.85; 0.96 | .90 | 0.23 | -0.54; 1.00 | .56 | -0.05 | -1.35; 1.24 | .93 |
| Group difference (intervention vs control) | 1.01 | -1.60; 3.61 | .43 | 1.86 | -0.76; 4.48 | .16 | 1.95 | | -0.30; 4.19 | .09 | 1.16 | -1.32; 3.64 | .36 | 2.15 | 0.28; 4.01 | **.02** | -0.94 | -4.35; 2.47 | .58 |
| Time effect only |  |  |  |  |  |  |  | |  |  |  |  |  |  |  |  |  |  |  |
| 3 months | -0.17 | -0.73; 0.39 | .54 | -0.10 | -0.75; 0.56 | .77 | 0.22 | | -0.45; 0.89 | .52 | -0.36 | -0.92; 0.20 | .20 | -0.15 | -0.63; 0.34 | .55 | -0.04 | -0.78; 0.70 | .91 |
| 12 months | -0.51 | -1.15; 0.14 | .12 | -0.03 | -0.83; 0.76 | .94 | 0.30 | | -0.59; 1.19 | .50 | -0.53 | -1.15; 0.10 | .10 | -0.36 | -0.94; 0.21 | .21 | 0.30 | -0.72; 1.33 | .55 |
| **eTable eTable 3. Waist circumference, BMI and metabolic syndrome Z-score after six and twelve months of lifestyle intervention in SMI patients stratified for gender, age group  and type of facility. Results of linear mixed models analyses with adjustment for AP medication side effect** (*continued)* | | | | | | | | | | | | | | | | | | | |
|  | **METABOLIC SYNDROME Z-SCORE** | | | | | | | | | | | | | | | | | | |
|  | **Gender** | | | | | | **Age** | | | | | | | **Facility** | | | | | |
|  | Males  (n=61) | | | Females  (n=47) | | | ≤46 yrs  (n=55) | | | | >46 yrs  (n=60) | | | F-ACT  (n=79) | | | Sheltered  (n=36) | | |
|  | **β** | **95% CI** | ***p*** | **β** | **95% CI** | ***p*** | **β** | **95% CI** | | ***p*** | **β** | **95% CI** | ***p*** | **β** | **95% CI** | ***p*** | **β** | **95% CI** | ***p*** |
| Intervention effect^a^ |  |  |  |  |  |  |  |  | |  |  |  |  |  |  |  |  |  |  |
| at 3 months^b^ | -0.22 | -0.87; 0.44 | .49 | -0.65 | -1.72; 0.42 | .14 | -0.31 | -0.84; 0.22 | | .25 | -0.45 | -1.03; 0.13 | .09 | -0.37 | -0.93; 0.19 | .18 | -0.03 | -0.17; 0.11 | .67 |
| at 12 months^b^ | -0.35 | -0.88; 0.19 | .19 | -0.52 | -0.98; -0.06 | **.03** | -0.35 | -0.92; 0.22 | | .21 | -0.51 | -0.92; -0.09 | **.02** | -0.13 | -0.58; 0.32 | .57 | -0.28 | 0.81; 0.26 | .31 |
| Group difference (intervention vs control) | 0.08 | -0.42; 0.57 | .75 | -0.14 | -0.72; 0.43 | .62 | 0.09 | -0.71; 0.88 | | .81 | -0.21 | -0.75; 0.33 | .45 | -0.16 | -0.83; 0.52 | .63 | -0.15 | -0.69; 0.38 | .57 |
| Time effect only |  |  |  |  |  |  |  |  | |  |  |  |  |  |  |  |  |  |  |
| 3 months | -0.04 | -0.47; 0.40 | .85 | 0.56 | 0.00; 1.11 | .050 | 0.02 | -0.30; 0.34 | | .90 | 0.60 | 0.27; 0.93 | **.01** | 0.26 | -0.15; 0.66 | .19 | 0.12 | 0.02; 0.23 | **.02** |
| 12 months | -0.01 | -0.35; 0.34 | .98 | 0.41 | 0.06; 0.76 | **.03** | 0.14 | -0.30; 0.59 | | .52 | 0.30 | 0.06; 0.55 | **.02** | -0.09 | -0.41; 0.24 | .58 | 0.07 | -0.30; 0.45 | .69 |

Abbreviations: AP medication: antipsychotic medication; CI: confidence interval; F-ACT: Flexible Assertive Community Treatment, F-ACT teams offer community-dwelling patients care in their own living environment.

^a^ control group is reference.

^b^ group x time.
